# Environmental cleaning and disinfection: Sustaining changed practice and improving quality in the community hospital

**DOI:** 10.1017/ash.2022.257

**Published:** 2022-07-12

**Authors:** Michael F. Parry, Merima Sestovic, Christopher Renz, Abegail Pangan, Brenda Grant, Asha K. Shah

**Affiliations:** 1 Division of Infectious Diseases, Department of Medicine, Stamford Health, Stamford, Connecticut; 2 Infection Prevention Department, Stamford Health, Stamford, Connecticut; 3 Environmental Services, Stamford Health, Stamford, Connecticut; 4 Vagelos Columbia College of Physicians and Surgeons, New York, New York

## Abstract

**Objective:**

Short-term improvements in hospital room cleaning can readily be achieved but are difficult to maintain. This is particularly true for high-risk, “high-touch” surfaces. Therefore, we embarked on a process to sustain improvements in surface cleaning and disinfection to reduce hospital-acquired infection (HAI) rates.

**Interventions:**

Our environmental services (EVS) and infection prevention departments incorporated a formal education, monitoring, and feedback process for focused cleaning and disinfection of high-touch surfaces into their routine policies and procedures in 2011. Cleaning validation was performed by infection prevention liaison nurses using a fluorescent targeting method to evaluate the thoroughness of cleaning.

**Results:**

Surface cleaning performance on medical-surgical units in 2011 was 74.7%, but this rate incrementally increased in response to the interventions and has been sustained at >90% for the past 6 years. Similar patterns of improvement were observed in the operating room, labor and delivery, endoscopy suite and cardiac catheterization laboratory. Conversely, HAI rates, particularly *C. difficile* rates, decreased by 75% and surgical site infection rates decreased by 55%.

**Conclusions:**

EVS training, monitoring, and feedback interventions, instituted 10 years ago have enhanced our environmental cleaning and disinfection efforts in multiple areas of the hospital and have been sustained to the present. Although other concurrent initiatives to reduce infection rates also existed, the improvements in environmental cleaning were associated with dramatic reductions in HAI rates over the 10-year period.

The cleanliness of a hospital room is an important determinant of an infection-free hospital stay. Pathogenic microorganisms can be repeatedly cultured from surfaces within patient rooms and many (eg, *Clostridioides difficile* or *Acinetobacter baumannii*) have been shown to survive on these surfaces for weeks to months.^1–3^ A patient admitted to a room where the previous occupant was colonized with a multidrug-resistant organism (MDRO) is likely to acquire the same organism, emphasizing the critical role of thorough room cleaning and disinfection in reducing hospital-acquired infections (HAIs), particularly due to MDROs.^4,5^ Furthermore, environmental pathogens contaminate healthcare workers’ hands, gowns, and gloves in proportion to the number of contaminated room surfaces.^2,6,7^ Despite these risks to patients and staff, studies of routine hospital environmental cleaning practices have found that historically less than half of hospital room surfaces are adequately cleaned and disinfected.^4,8–11^

Considerable efforts have been made to improve the quality of cleaning in the patient’s environment through focused education and monitoring housekeeper performance.^8,12–15^ Implementation of such strategies in real-world settings, however, has been difficult, and considerable variability remains in cleaning practices by environmental services (EVS) staff.^10,16,17^ Several studies have documented short-term improvements through the use of objective cleaning measurements, such as adenosine triphosphate (ATP) testing or removal of fluorescent markers, but sustainability has either not been measured or performance has deteriorated at the conclusion of the formal intervention period.^13,18–21^

Given these concerns and the positive results published in short-term environmental cleaning studies, we integrated the lessons learned from these studies into our EVS policies and procedures. We created a formal curriculum and monitoring program using a fluorescence targeting method to evaluate the thoroughness of patient room cleaning. Our report documents the program’s sustainability over a 10-year period, its expansion to other patient-care areas, and its impact on hospital-acquired infection rates.

## Methods

Stamford Hospital is a 305-bed community hospital with a primary service population of ∼150,000 people in southwestern Connecticut. The facility existed as a mix of private and semiprivate rooms until September 2016, when all medical-surgical units and the intensive care unit (ICU) moved into a new tower with all single-patient rooms.

Our initial efforts in environmental cleaning were a series of short, prospective trials to evaluate the thoroughness of terminal room cleaning and disinfection over a 3-year period, previously published.^8,10,20^ Trials spanned the years 2006–2009, using convenience samples of rooms on the adult general medical-surgical units and ICU with adjoining bathrooms. EVS leadership, with input from the infectious diseases team, provided a standardized educational program for both line and supervisory EVS personnel. The program reviewed the importance of disinfection and cleaning for the safety of patients, visitors, environmental services personnel, and other healthcare workers; it clarified the importance of cleaning “high-touch” surfaces; and it provided a hands-on demonstration of the evaluation system while emphasizing the importance of the housekeepers’ work in the overall infection prevention activity of the institution. Feedback was provided to the EVS staff in both group and one-on-one teaching sessions to optimize terminal, daily, and between-case cleaning activities in the operating room. Such activities were complemented by active infection preventionist rounding and (prior to its suspension due to the COVID-19 pandemic) a formal biennial infection prevention coach training program that provided additional education and coach skills training to all staff, including EVS employees. Expansion of the program from the originally designated ICU and medical-surgical units included the main operating rooms (January 2014), cardiac catheterization laboratory (January 2017), labor and delivery suite (July 2017), specialty operating rooms (October 2017), and the endoscopy suites (February 2018).

In January 2011, EVS incorporated the education and monitoring process into its routine policies and procedures. In 2014, the onboarding process for EVS aides was restructured. Newly hired staff members were paired with more advanced aides and were required to work a minimum of 3 paired shifts on inpatient or invasive surgical area assignments. During these 3 shifts, newly hired aides were required to show competencies in daily and discharge cleaning, along with in-between case cleaning of invasive areas. The advanced EVS aide validated the newly hired aide’s knowledge of the cleaning process and validated their competency. All EVS personnel are reassessed annually for their room-cleaning skills and are observed by EVS leadership to ensure competency.

To evaluate the thoroughness of room cleaning and disinfection, select nursing personnel, referred to as infection-prevention liaison nurses, were trained to evaluate EVS performance. This unique cadre of nurses was chosen from each patient-care area for their expressed interest in infection prevention. Their role was incentivized by incorporation into the nursing clinical ladder for professional growth and financial reward. They represent extensions of the infection preventionists as behavioral coaches in promoting staff immunization, hand hygiene, infection surveillance and MDRO management in addition to environmental cleaning. With their extra training and expertise, they are able to assess the adequacy of cleaning and to engage the EVS staff and leadership to maximize their performance. The teamwork in this initiative has been viewed as a priority by hospital administration for patient and staff safety.

Infection-prevention liaison nurses mark and evaluate cleaning of surfaces in the patient care environment using a transparent, easily cleaned marking gel that fluoresces when exposed to ultraviolet light (Dazo^TM^, Ecolab, St. Paul, MN). Furthermore, 13 predefined high-touch surfaces (Table 1) were marked in each patient room and were then evaluated for thoroughness of removal after the EVS staff had cleaned the room. The outcomes were categorized as “cleaned” or “not cleaned.” When most of the fluorescent marker had been removed but small amounts of smeared fluorescence were still detectable, credit was given as “cleaned.” Variations in the room structure and equipment from floor to floor sometimes required alternate surfaces be used to achieve an average of 13 sites per room. Similarly, surface marking in the operating room and special procedure rooms required different designated sites owing to different room designs and functions. Medical-surgical rooms were selected at random by nursing staff, based on admission and discharge demands and nursing time available to perform fluorescent gel marking and reading. Surfaces were marked by nursing staff without advance notification of the housekeeper. The medical-surgical unit goal was a minimum of 4 rooms per patient-care unit (52 surfaces) per month. Similar goals were set for other areas as they were added to the program.

**Table 1.** Designated High-Touch Surfaces for Fluorescent Marking in Patient Rooms on the Medical and Surgical Floors

Surface location
Toilet seat
Toilet flush handle
Sink top
Bathroom door handle
Bedside rail
Telephone
Room counter workspace
Room cabinet handle
Call bell/TV controller
Tray table
Patient chair arm
Room door handle
Computer keyboard

Fluorescent gel was applied using a single-dose, sponge-tipped 2-mL applicator for all sites in each room by the nursing staff. The gel was invisible after drying, achieving as close to a blinded evaluation of cleaning as possible. Removal was assessed by the same nurses using a handheld ultraviolet fluorescent light (mini-blacklight-2, China). If >3 surfaces in a room were documented to have failed (ie, were uncleaned), the EVS supervisor was notified, and the room was closed and recleaned prior to patient occupancy. Cleaning was performed with a variety of products over time to achieve a combination of cleaning and disinfection on all surfaces (most recently, OxyCide^TM^, Ecolab, St Paul, MN).

### Data and statistical analysis

Nurses recorded the results for each surface and room on a spreadsheet and forwarded them to infection prevention personnel for monthly aggregating and tabulating and to the EVS manager for education and re-education of staff. Collated results were presented to the infection prevention committee every month and were reviewed by the EVS manager for any required remedial training. Unit-based results were posted electronically on the Infection prevention intranet site and were provided to the unit managers in a standardized graphical format. Annual HAI, surgical site and *C. difficile* infection rates were determined using standard Center for Disease Control (CDC) and National Health and Safety Network (NHSN) definitions.

## Results

**Fig. 1.**
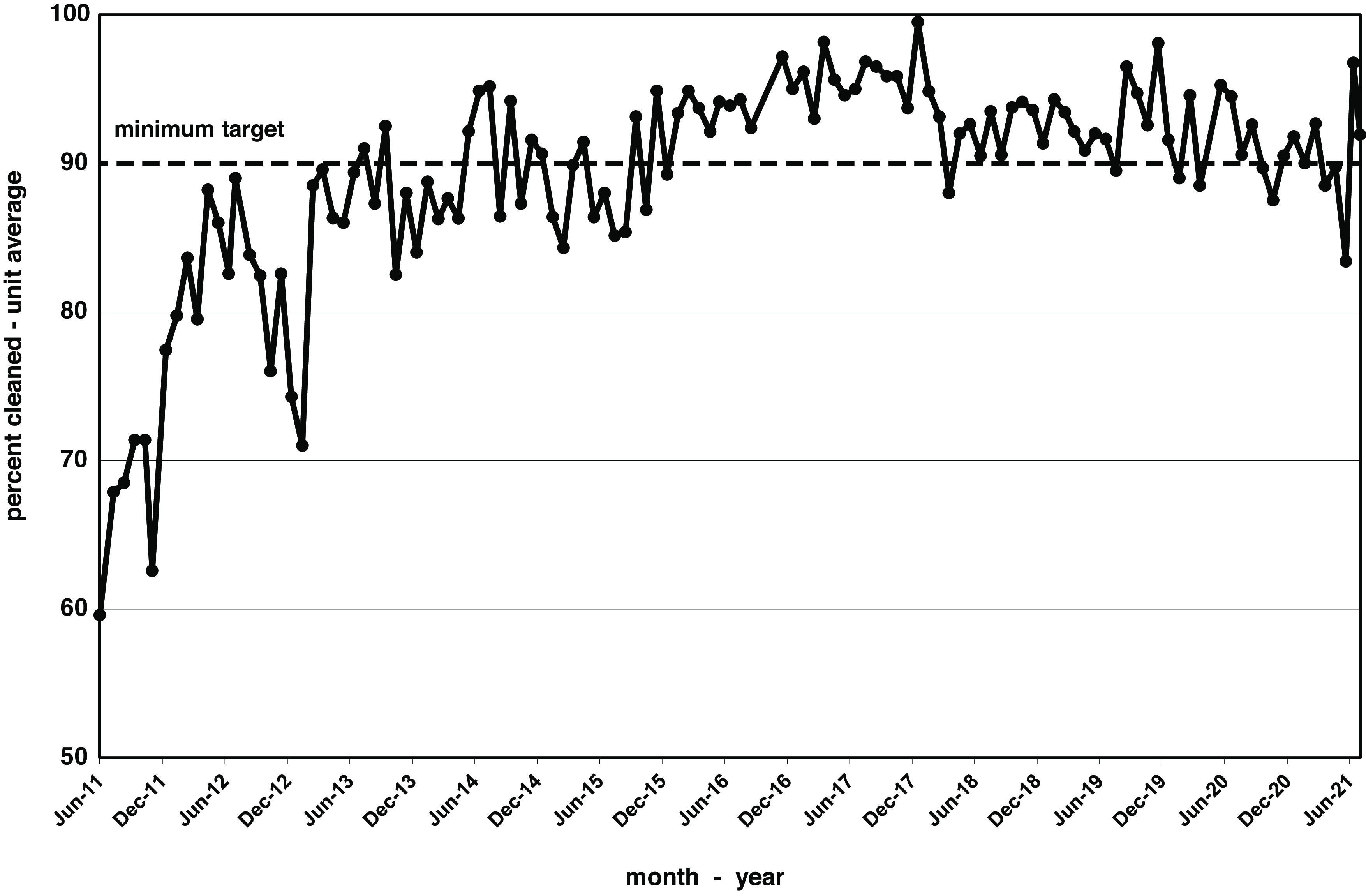
Monthly cleaning rates over 10 years on medical and surgical floors assessed as a percentage of all surfaces cleaned.

Environmental cleaning and monitoring results reporting for the medical-surgical units began in June 2011. Preliminary cleaning data were similar to frequencies noted in our older studies at 60%–70% after short-term interventions.^8,10,13^ It took 4 years of ongoing improvement to achieve cleaning rates that met our 90% target. Once this level was achieved, the annual cleaning performance continued to be >90% for the next 6 years. Figure 1 displays monthly cleaning rates over the 10-year period from June 2011 to June 2021, or 117 months. However, 3 months were excluded due to lack of data: 2 at the time of transition from the old hospital building to the new building in September and October of 2016 and 1 at the peak of COVID-19 cases in April 2020. Cleaning rates averaged 74.7% for the first year but reached 90% by year 4 and remained >90% in the following years. Cleaning has averaged 93.0% over the past 5 years, prompting revision of the cleaning target to 93%.

Fluctuations in performance often reflected changes in leadership. New EVS leadership came on board in March 2014, refreshed the training program, and clarified the expectations of high compliance. Deficiencies in individual housekeeper performance were directly addressed by the new leadership. Housekeeper re-education, especially the relevance of high-touch surface cleaning and disinfection, mentorship, performance feedback, personnel reallocation and hiring additional full-time equivalent housekeepers were all used when appropriate. A transition in infection prevention leadership and relative staff shortages due to the COVID-19 pandemic in 2020 and 2021 led to a slight decline in cleaning rates.

Our goal was to monitor 13 sites in 4 rooms per patient-care unit per month, providing 4,992 surface tests per year. We achieved 80% of this goal. Of 8 medical-surgical units involved in the program, an average of 6.94 units reported data every month.

**Fig. 2.**
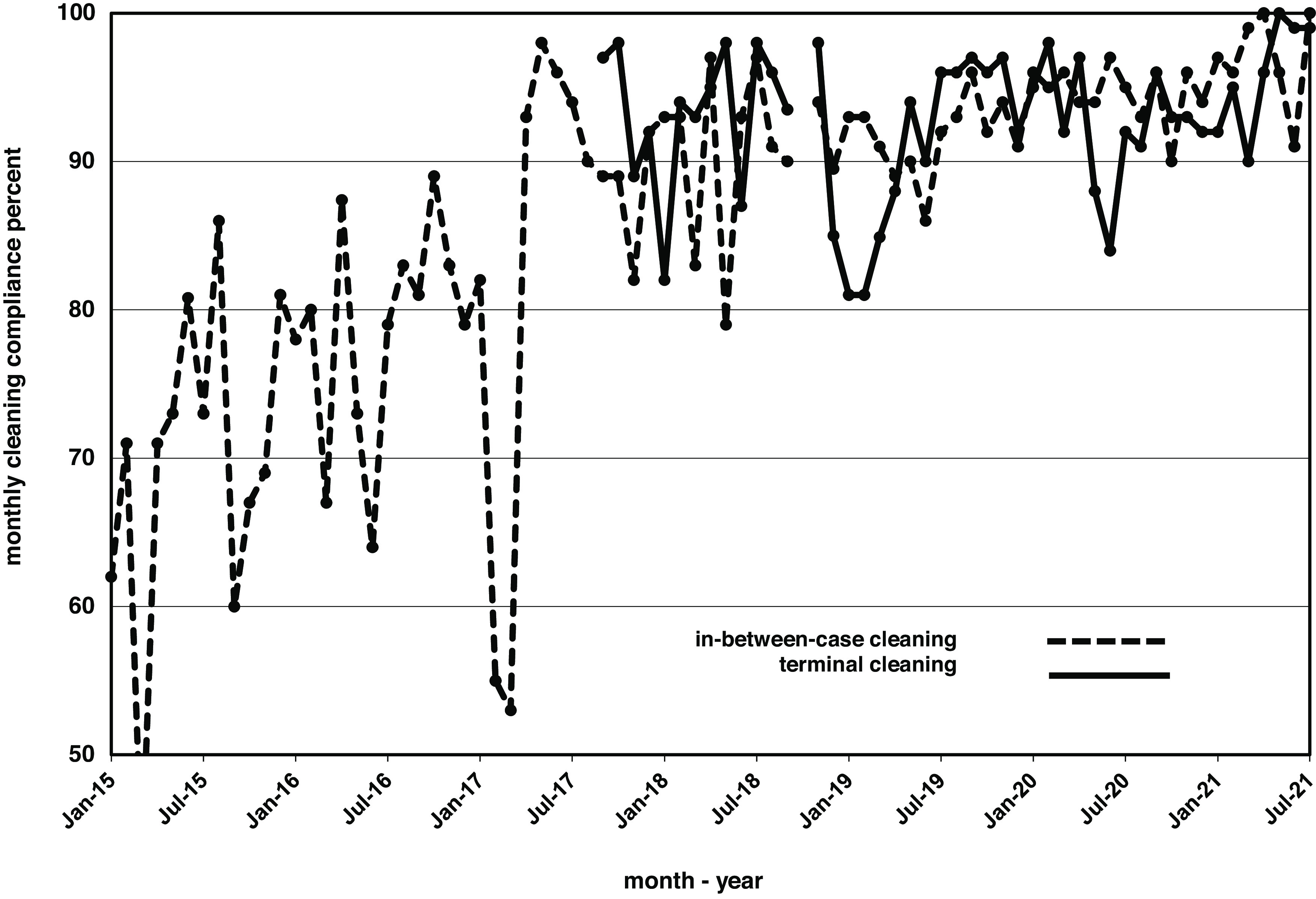
Monthly cleaning rates, measured as the percentage of surfaces cleaned, in the main operating rooms for terminal cleaning (solid line) and between-case cleaning (dotted line).

**Fig. 3.**
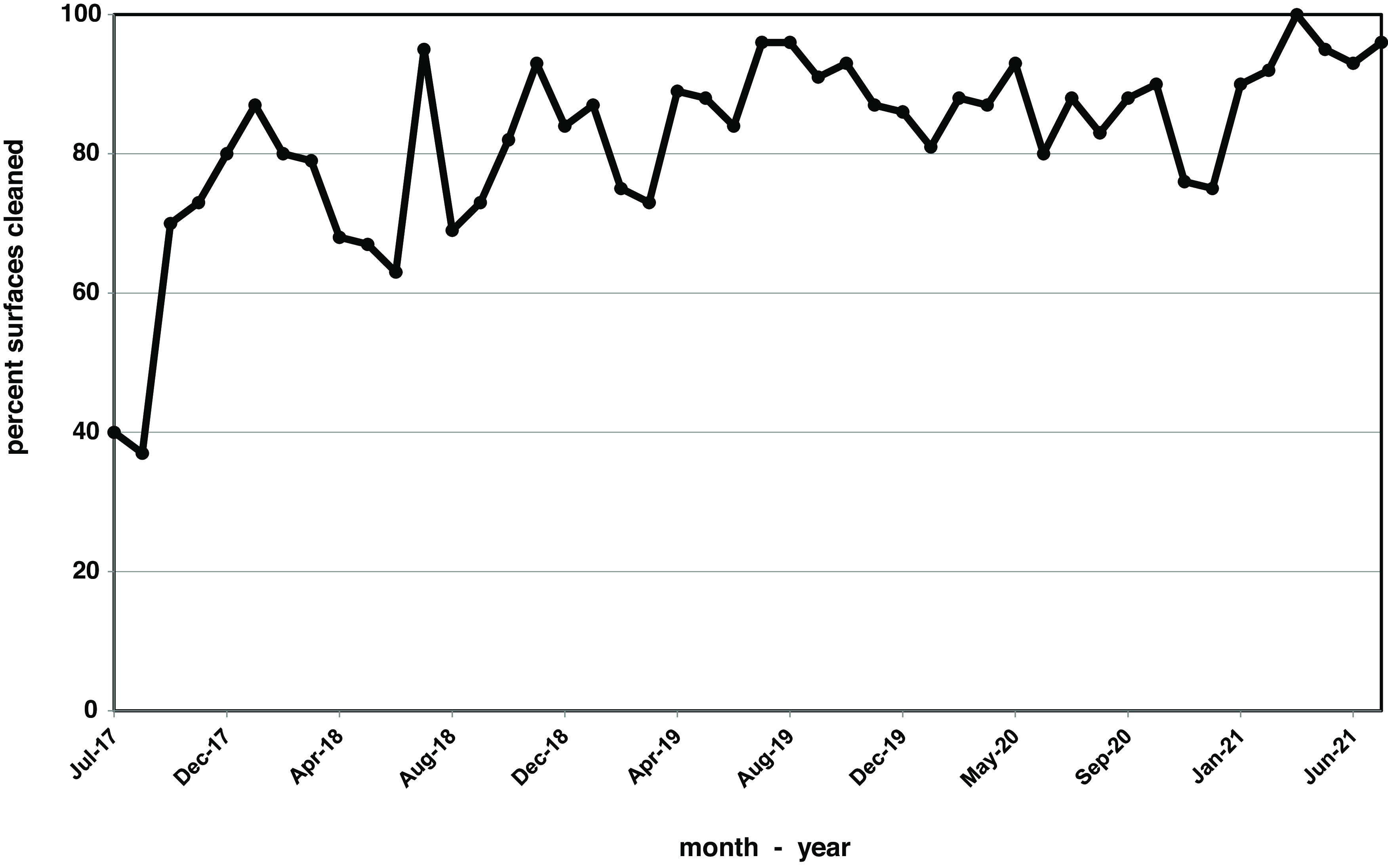
Monthly cleaning rates, measured as the percentage of surfaces cleaned, in labor and delivery.

After 3 years of measurable improvement on the medical-surgical units, we allocated resources to expand the program to other patient-care areas. The main operating room came online in January 2014; however, due to personnel issues, monthly reporting did not begin until January 2015, initially for between-case cleaning, followed by terminal cleaning (Fig. 2). Cleaning progressively improved and consistently met or exceeded the 90% target in 22 of the 24 measures over the past 2 years. These gains were subsequently duplicated in other areas, such as labor and delivery (Fig. 3).

**Fig. 4.**
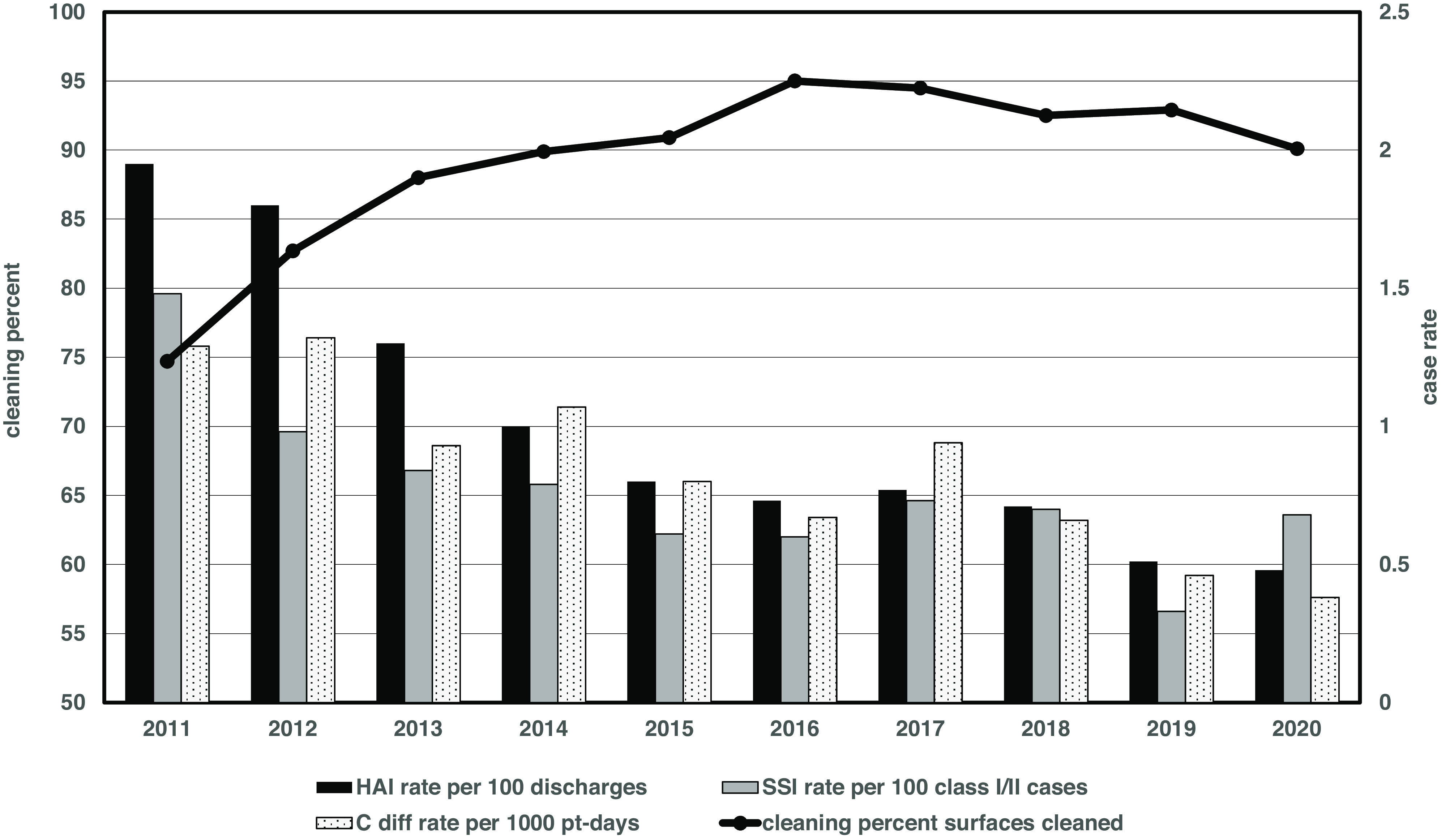
Improvement in environmental cleaning and declining rates of hospital-acquired infections (HAI), class 1 and 2 surgical site infections (SSI), and hospital-onset *C. difficile* infections (CDI) over the 10-year study period, 2011–2020. HAI and SSI rates were determined according to National Health and Safety Network (NHSN) definitions. CDI rates were determined by NHSN criteria for laboratory determined (LABID) events but were adjusted to 1,000 patient days rather than 10,000 patient days for graphical simplicity.

Improvements in cleaning (and disinfecting) performance throughout the hospital were associated with a 10-year decline in overall hospital-acquired infection rates (by 75%), surgical site infection rates (by 55%), and rates of hospital-acquired *C. difficile* (by 70%) (Fig. 4). The reduction in *C. difficile* infection rates is particularly noteworthy because of its environmental persistence. However, numerous other initiatives coexisted making a univariate analysis impossible. Other initiatives included process improvement projects for CLABSI, CAUTI, and SSI rate reduction, an ongoing hand hygiene compliance program and various antibiotic stewardship initiatives. Additional EVS enhancements were also underway during this time, including revision of our housekeeping mentorship process, expansion of OxyCide disinfection (from a combination of bleach and quaternary ammonium products), and the addition of an ultraviolet light program (Xenex Disinfection Services, San Antonio, TX) to enhance terminal disinfection.

## Discussion

Often overlooked in preference to novel improvement program initiatives, basic environmental hygiene remains a vital component of the infection prevention tool kit. Lapses in environmental cleaning and disinfection in healthcare facilities are common despite national guidelines and individual institutional policies, partly due to the lack of consistent and ongoing monitoring of the thoroughness of cleaning practices. Our experience shows that long-term, sustained improvement in environmental cleaning and disinfection can be achieved with programmatic changes in EVS department work processes and inspirational leadership. Only 40%–50% of high-touch surfaces were consistently cleaned prior to intervention, but this rate rose to 60%–70% after short-term-study interventions and finally reached sustained levels of >90% for the past 6 years. Others have achieved similar consistency by EVS education, behavior change, objective measurement, and consistent, impartial feedback on individual EVS employee performance.^12,21,22^

Performance monitoring cannot be delegated to EVS staff themselves, although education and mentorship require EVS leadership to play a role. When our infection prevention liaison nursing team temporarily relinquished the role of monitoring in 2009–2010, spot checks revealed that surface cleaning had actually dropped to 50%, whereas EVS reported compliance rates of 90%–100%. These elevated cleaning rates were presumably motivated by focusing on the report card; “telegraphing” the location of fluorescent targets to facilitate their identification and focused cleaning rather than thorough cleaning; or surreptitious use of a black light by individual housekeeping staff. Other researchers have noted the same tendency.^15^ For example, Knelson, et al^23^ found that in rooms tested by EVS supervisors, 82.5% of high-touch surfaces were found to be clean, whereas in rooms tested by study personnel, only 52.4% of surfaces were found to be clean (*P* < .001). Discrepancies were greater for some surfaces (door knobs and light switches, 92.6% vs 23.5%) than others (eg, sink, toilet seat, and toilet hand rail). Therefore, all our EVS cleaning assessments were performed in rooms randomly selected and interpreted by our specially trained infection-prevention liaison nursing staff.

The EVS training program focused on the importance of “high-touch surface” cleaning and disinfection. Even at the beginning of training, when our average cleaning rate was 45%, the sink, toilet seat and toilet hand rail were cleaned >75% of the time, but the telephone, door knob, light switches, and call bell were only cleaned an average of 30% of the time.^8^ With education, high-touch surface cleaning rapidly improved.^12,20^ Ongoing, focused, remedial training is enabled by monthly review of performance data to address deficiencies in cleaning specific items or in specific patient-care areas. Corrective actions can then be taken in a timely manner.

At inception of the program, EVS aides were often defensive about being shown missed areas of cleaning. On many occasions, the EVS aide felt accused, stating, “I did clean this area; the nurse must have placed the ‘goo’ on the spot after I was done with my cleaning.” As EVS leaders and infection preventionists worked alongside the EVS aides, mentoring, and rewarding good performance, the EVS aides took ownership and pride in scoring 100% in cleaning validation. Now “high-touch-point cleaning” is the cornerstone of our overall cleaning and disinfection process, and the EVS aides are keenly aware of their role in keeping patients free from infection. Frontline housekeepers now work more closely with nursing staff, and many have assumed a coaching role as part of the infection prevention team.

Our initial focus on patient room cleaning and disinfection was based on the patient room assignments and the common physical design of regular patient rooms. However, specialty care units present significant risks that are inherent to the procedures performed there. Like others, we have expanded our focus to 4 areas: the operating room, the labor and delivery suite, the cardiac catheterization laboratory, and the endoscopy suite.^9,24^ Extension of the cleaning validation process to these areas represented a challenge, requiring incorporation of both between-case cleaning and terminal cleaning, as well as selection of alternate high-touch surfaces. However, using the same process of nursing validation after EVS cleaning, we successfully implemented the process on 2 hospital campuses, and we have sustained cleaning rates of >90% in all 4 areas (Fig. 3). Other high-risk areas, such as interventional radiology, wound care centers, and infusion centers could be incorporated into the process and are under discussion.

We have continued to use our predetermined 13 high-touch surfaces in patient-care rooms, with minor variations. However, others have shown that a smaller number of sites can be used effectively.^25^ Although sites are somewhat different in procedural areas, we mirrored the number of surfaces tested, varying between 10 and 16 per room, or 50–75 surfaces per month. We have also maintained the use of the same consistent marking process using the fluorescent gel originally developed by Carling.^8^ Other investigators have used fluorescent powder or adenosine triphosphate (ATP) detection in a similar fashion.^26–28^

Neither fluorescent gel, fluorescent powder, nor ATP removal measures disinfection. In our institution, all cleaning is performed with a cleaner-disinfectant combination, so we expect that surface disinfection will be accomplished by the cleaning process. Conflicting data exist to support this expectation. Some have found that cleaning does not accurately predict disinfection, as determined by surface microbial colony counts, even if antimicrobial products are used.^3,29,30^ We did not specifically measure disinfection, but over the 10-year period of improving environmental cleaning, we experienced a 75% reduction in overall hospital-acquired infection (HAI) rates, 55% reductions in class I and II surgical site infection (SSI) rates, and a 70% reduction in hospital-onset *C. difficile* rates. Other studies have also shown a reduction in HAIs with enhanced cleaning and associated disinfection.^31–39^

The limitations of our study are, in some ways, its strength. We did not attempt to control room selection or rigidly monitor participant (nursing or housekeeping) activities aside from ongoing education and regular performance feedback. Our interventions were incorporated into daily hospital practice. Second, except for between-case cleaning in the operating rooms, the process focused on terminal cleaning rather than daily cleaning. We hoped that the feedback provided, and the improvements measured, would translate into the quality of daily cleaning, but this was not measured. Third, we measured cleaning, not disinfection. As noted, these do not always coincide. Nevertheless, our results suggest that surface disinfection was effective in decreasing infection rates throughout the hospital. Fourth, our hospital is unique in being a community hospital with excellent staffing, supportive leadership, and all single rooms. Our results may not be representative of other institutions. Finally, over the decade of analysis, we participated in multiple other efforts that we would expect to have improved infection rates, including quality improvement programs in hand hygiene, antibiotic stewardship, CLABSI, CAUTI, and SSI reduction. The specific contributions of environmental cleaning and disinfection cannot be fully isolated from these other initiatives.
